# Tattooing to reconstruct Nipple-Areola Complex after oncological breast surgery: a scoping review

**DOI:** 10.1007/s00520-024-08351-3

**Published:** 2024-02-10

**Authors:** Deborah Maselli, Martina Torreggiani, Tiziana Livieri, Gloria Farioli, Stefania Lucchi, Monica Guberti

**Affiliations:** 1 International Doctorate School in Clinical and Experimental Medicine, Università Degli Studi Di Modena E Reggio Emilia, Modena, Italy; 2 Azienda USL-IRCCS of Reggio Emilia (Health Professions Department), Reggio Emilia, Italy; 3 Azienda USL-IRCCS of Reggio Emilia, Reggio Emilia, Italy

**Keywords:** Medical tattooing, Women health services, Breast cancer nursing, Oncology, Nipple-Areola complex reconstruction

## Abstract

**Purpose:**

The dermopigmentation of the Nipple-Areola Complex (NAC) is a safe non-surgical reconstruction technique that can restore psychophysical integrity, representing the final step after oncological surgery. This scoping review aims to identify and synthesize the literature focused on medical tattooing for NAC reconstruction in women who underwent breast reconstruction after cancer surgery. Competence and training, outcomes and organizational aspects were assessed as specific outcomes.

**Methods:**

The Joanna Briggs Institute (JBI) methodology for scoping reviews was followed. MEDLINE, Embase, Cochrane Library, Clinical Key, Scopus and Cinahl databases were consulted. After title (*N* = 54) and abstract (*N* = 39) screening and full-text review (*N* = 18), articles that met eligibility criteria were analyzed**,** critically apprised and narratively synthesized.

**Results:**

13 articles were analysed, with full texts (*N* = 11) and only abstract (*N* = 2). The overall quality of the literature (N observational studies = 11; N pilot experimental studies = 2) is weak. Nurses were the professionals mostly involved (*N* = 6), then medical staff (*N* = 4) and tattoo artists (*N* = 2). The professional training is poorly described in 6 papers. The most frequently assessed outcome was the satisfaction rate (*N* = 8). One study explored aspects of quality of life with a validated questionnaire. The management of these services resulted variable. Nurse-led services were implemented in 2 studies.

**Conclusion:**

Despite methodological weaknesses, NAC tattooing research is relevant because it helps women redefine their identity after demolitive cancer treatments. Further research on processes and outcomes is needed.

## Introduction

At the end of 2020, breast cancer was the most prevalent cancer in the world [1]. If the tumor position is found in the tissues below the areola or on the surrounding skin**,** Nipple-Areola Complex (NAC) removal may be necessary. This procedure can compromise the aesthetic result of breast reconstruction and have a substantial psychological impact on the patient [2]. The NAC medical tattooing, or dermopigmentation, is a simple and safe non-surgical reconstruction technique that leads to recovery by restoring the psychophysical integrity [2]. It can reduce costs, waiting times, and related complications compared to surgical procedures. High satisfaction levels of the aesthetic results are also demonstrated [3]. The tattoo can be performed alone or with other reconstruction techniques, such as skin grafts, to integrate color and appearance [3–6].

Moreover, it represents the only possible option in case of some contraindications (damaged tissues, comorbidities, anxious states related to past hospital experiences) [7, 8]. Rees initially introduced the technique in 1975 [9], and it has been recently refined [10, 11]: the sterile/semi-sterile skin pigmentation is performed by a specifically trained professional through the introduction of natural pigments into the superficial papillary dermis with a disposable needle, through a dermographer or manually [12]. The experiences reported in the literature differed in professional and management aspects according to healthcare contexts. In Italy, this technique is usually performed by professional tattoo artists [8], with considerable costs for patients. Nevertheless, some encouraging experiences of in-clinical services are growing as the final part of the breast reconstruction after cancer surgery and treatment clinical pathway [8, 13]. NAC dermopigmentation is included in the essential levels of assistance guaranteed by the National Health Care System, free of charge [2]. The implementation of nurse-led multidisciplinary services can be cost-effective (no operating room is needed) [2, 3].

Further, this service can represent a familiar patient referral through the therapeutic relationship with the clinical nurse specialist [14, 15]. Nurses are essential in supporting women in making informed decisions about all aspects of their care [16]. Until 2010, only a handful of small studies examined patient satisfaction following NAC tattoo reconstruction, as they were relatively the first experiences studied [17]. After a preliminary search of MEDLINE and the Cochrane Database of Systematic Reviews, two narrative reviews investigating the tattoo techniques and theories were found, with poor robustness in methodology and quality assessment of evidence [3, 8]. How this intervention impacts women’s lives, healthcare organizations, and professional competence is unclear. No systematic or scoping review on the topic was found. This scoping review aims to identify and synthesize the literature focused on medical tattooing for NAC reconstruction in women who underwent breast reconstruction following breast cancer surgery.

## Methods

A scoping review methodology was chosen, as it provides a literature mapping on a specific topic, showing relevant concepts, gaps in the research, and types of existing evidence [18]. This review will gain the general objective by answering the following primary research question: what is the evidence about medical tattooing for the NAC reconstruction in breast cancer patients who underwent surgical treatments? Additionally, specific outcomes will be achieved by answering the following secondary research questions:

1. What type of professionals, competence, and training are involved in providing the intervention?
2. Which outcomes and/or Patient Reported Outcomes (PROs) are assessed in the studies?
3. How is this intervention delivered in the healthcare settings considered by the studies?

The proposed scoping review was conducted following the Joanna Briggs Institute (JBI) methodology for scoping reviews [19]: the process started with defining the objective and question, followed by developing the inclusion criteria, evidence research following the planned strategy, selection, and extraction of the evidence, analysis, presentation, and summarizing the evidence. This scoping review was reported by the Preferred Reporting Items for Systematic Reviews and Meta-Analyses extension for Scoping Reviews (PRISMA-ScR) checklist [20].

### Search strategy

The PICO method (P = population, I = intervention, C = control, O = outcome) was used to make the research question. The women who underwent breast reconstruction following breast cancer surgery treatment were included in the population frame; the intervention considered was the tattoo performed to reconstruct the NAC; no control was assessed; outcomes are described above. A literature search was conducted within the following databases: Pubmed, Embase, Cochrane Library, Clinical Key, Scopus, and Cinahl. The main keywords “*Nipple-Areola Complex*” and *“tattoo*” were used in Pubmed and also adapted in the other databases with the keywords “*nurse*”, “*breast*” and “*dermopigmentation*”. The records were filtered by time frame (from 01/01/2010 to 28/02/2023) and involved subjects (Human). Reference lists of included papers were manually checked to find further records.

### Eligibility criteria

Studies that considered the intervention in association with other techniques for NAC reconstruction were included, as well as those evaluating staff perceptions, other organizational outcomes, and any measurable change in health conditions. This review considered complete and primary published literature: all experimental study designs, prospective and retrospective cohort studies, case–control studies, analytical cross-sectional, descriptive observational study designs including case series, and descriptive cross-sectional studies. Qualitative studies were also evaluated. Exclusion criteria were listed as follows: non-English languages; incomplete or unpublished literature, conference papers, case reports, and theoretical/position papers. We excluded papers that described other reconstruction methods alone. Finally we excluded studies that explored the tattooing technique in other regions different from the NAC, with a non-reconstructive aim (for example, decorative/artistic tattoos), and those involving non-oncological patients.

### Study selection

**Fig. 1:**
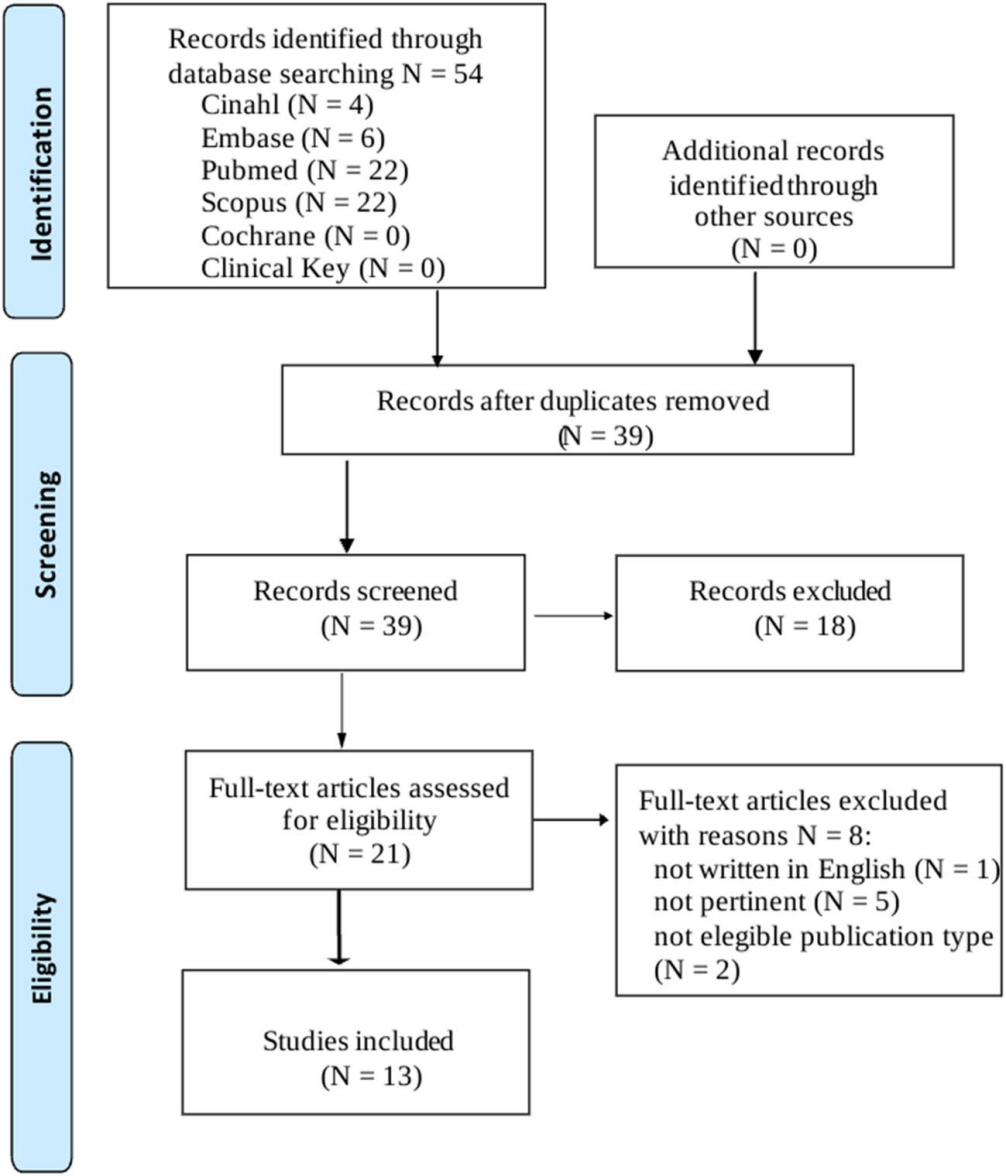
PRISMA Flow Diagram for the scoping review process

The study selection process is presented in a Preferred Reporting Items for Systematic Reviews and Meta-analyses extension for scoping review (PRISMA-ScR) flow diagram (Fig. 1) according to the PRISMA-ScR statement [20]: the search results were imported into Mendeley software and screened for eligibility by two independent reviewers in each phase. Duplicates, as well as non-relevant records, were manually removed in two steps: firstly, evaluating the title and the abstract, and secondly, after the full-text reading. Any reviewer disagreements were resolved through discussion at each stage of the selection process.

### Data extraction

One reviewer manually extracted the data from the selected articles using Excel, which was cross-checked independently by another review team member. Any disagreement identified was resolved through discussion with other review team members. If full texts were unavailable, the abstracts were considered in the analysis, reported, and discussed separately. Extracted data included author, year, Country, population, methods, adverse events, and main results. These elements were sought according to the specific outcomes stated before. A narrative and thematic synthesis was conducted to summarize the results.

### Critical appraisal of included literature

Studies were critically evaluated according to the Equator network’s reporting guidelines [21]. Secondly, a more profound quality assessment was conducted with the Effective Public Health Practice Project (EPHPP) [22]: for clinical trials, randomized clinical trials (RCTs), and observational studies, it is a widely used instrument with an excellent degree of inter-rating reliability. The score’s sum of the six domains (selection bias, study design, confounders, blinding, data collection methods, withdrawals, and drop-outs) constitutes the overall quality rating, which can be “strong”, “moderate” or “weak”. Two independent reviewers conducted a quality assessment and discussed and solved any discrepancies. This step provided more certainty when referring to this intervention, specifically on how it should be methodologically evaluated and implemented in different healthcare contexts.

## Results

### Literature search results

As represented in Fig. 1, the literature search identified 54 records: 22 from Medline, 6 from Embase, 0 from the Cochrane Library, 0 from Clinical Key, 22 from Scopus and 4 from Cinahl. After removing 15 duplicates, 39 articles were screened by title and abstract reading. 21 papers were assessed for eligibility: 8 studies were excluded: not written in English (*N* = 1), not pertinent (*N* = 5) and not eligible publication type (*N* = 2). Finally, 13 articles were included in the review. Eleven studies included the full texts, while 2 were available as abstracts only.

### Characteristics of the included literature

The articles involved participants from Europe (*n* = 6), America (*n* = 4), Asia (*n* = 2) and Oceania (*n* = 1). Observational studies were the most frequent results (*n* = 11), while two were pilot experimental studies. Papers were classified by publication type (full texts or abstracts). Eight studies evaluated the satisfaction rate given by the patients, and three the aesthetic results, mostly considering appearance and symmetry [10], color match [23]; one study also evaluated the perceived usefulness of the NAC tattoo service [2]; one paper aimed to identify the risk factors for tattoo-related breast infections [13]; two studies focused on the relationship between radiotherapy and tattoo fading [24, 25]; one study involved healthcare personnel, determining if the likelihood of discussing options for NAC tattooing differed between Registered Nurses (RNs) and non-RNs [26].

### Quality assessment

As reported in Table 1, the overall quality of evidence is weak: most of the results were observational studies (based on medical records [13], with small samples [6], not methodologically rigorous [23, 25, 27]). Another limitation was found in the outcome measurement: none of the studies used validated instruments except one [28]. The experimental studies aimed to evaluate the technique’s safety and the intervention’s feasibility, with the need for larger and more structured projects [2, 11].

**Table 1:** Quality assessment of the included literature

Paper	EPHPP
Gava et al. (2020)	Moderate
Weissler et al. (2021)	Moderate
Uhlmann, Martins and Piato (2019)	Weak
Smallman et al. (2018)	Weak
DiCenso and Ficher-Cartlidge (2015)	Weak
Sowa et al. (2021)	Weak
Goh et al. (2011)	Weak
Cha et al. (2019)	Weak
Murphy et al. (2010)	Weak
Starnoni et al. (2020)	Weak
Aslam et al. (2015)	Weak

### Findings

Main findings are summarized in Table 2 and 3. In Sowa et al. [10], plastic surgeons retrospectively evaluated the NAC tattoo’s appearance and symmetry made with the 3D-E technique (*n* = 61) versus the conventional technique (*n* = 49) after flap-based nipple reconstruction in a Japanese facility: significantly higher ratings of appearance and symmetry were found in the 3D-E tattoos cohort. Gho et al. [17] retrospectively measured patients’ satisfaction with the NAC after tattooing among 172 women in a UK facility: 70% of responders (*N* = 110) were satisfied. In Italy, Starnoni et al. [27] measured the satisfaction levels among tattooed women after NAC reconstruction: 92% of 48 patients were satisfied or very satisfied. In a more extensive Italian pilot study [2], an advanced practice dermopigmentator performed NAC tattoos on 169 women who underwent surgical breast reconstruction: 90% of women were highly satisfied with aesthetic results, and 97% considered the project useful or very useful. In Brazil, Uhlmann et al. [11] tested 3D dermopigmentation technique for the NAC reconstruction, evaluating respectively the satisfaction of the professionals and the patients (*N* = 20): the first ones perceived as “good” or “excellent” the overall aesthetics (76%) and color (72%); patients indicated as “good” or “excellent” the overall satisfaction (95%) and color (100%). In Ireland [23], a clinical nurse specialist performed NAC tattoos on women with reconstructed breast: the median score of satisfaction with the color was 4.6/5, and the mean color match between the tattooed areola and the contralateral one was 91%. In Cha et al. [6], 20 women underwent a tattoo-only technique for NAC reconstruction after an oncological mastectomy performed by one plastic surgeon in a Korean medical facility: these patients mostly showed reluctance to another operation, skin problems, collateral effects of radiotherapy and chemotherapy, smoking habit; the average overall satisfaction score was 8.1 on a 10-point scale, significantly the highest compared with the other techniques one. In one Australian cohort study [28], women who underwent NAC tattoos were asked to express their opinion about satisfaction with the nipple, as well as quality of life, sexual well-being, and other psychosocial issues, with a validated questionnaire before and after the intervention. The authors also wanted to identify any differences in patient satisfaction between those treated by the nurse practitioner (*N* = 169) and those by the plastic surgeon (*N* = 111): among responders (48%), a significant improvement was found in the patient’s satisfaction with their NAC, but no significant differences were found between clinicians. Di Censo et al. [26] wanted to determine if the likelihood of discussing options for NAC tattooing and recommending tattoo artists differed between Registered Nurses (RN) (*N* = 43) and non-RNs (*N* = 25): no significant difference was found between the groups on awareness and recommendation of a tattoo artists providing a NAC tattoo. Aslam et al. [25] aimed to identify if radiotherapy exposition influenced tattoo fading among 292 women after flap reconstruction in a UK medical facility: the median time of fading was 4,5 months. 70% of the people who had radiotherapy and subsequent NAC tattoos had problems retaining the tattoo pigment and required repeated tattoo sessions. In the USA, an institutional review [13] of 539 patients who underwent NAC tattooing after reconstructive procedures showed that the tattoo-related infections were 2.2% (*n* = 21 breasts), while the mean time to infection was 6.5 days; also, 85.7% of infections occurred in Implant Based Reconstruction (IBR) patients, and one-third of them had previous radiation treatment. This study suggests that previous radiation and pre-pectoral IBR are independent predictors of tattoo-related breast infection. Finally, two abstracts were included in the analysis of the results (Table 3): Rider et al. [24] aimed to determine if the type of reconstruction [Transverse Rectus Abdominus Myocutaneous (TRAM) flap reconstruction (*N* = 31) and Latissimus Dorsi (LD) flap reconstruction (*N* = 93)] or the post-reconstruction RadioTherapy (RT) have any effects on the number of tattooing episodes required to obtain a satisfactory result: no significant difference was found between the number of patient satisfied after one or more session in all groups (LD no RT, LD with RT, TRAM). Finally, one study [29] quantified changes in NAC tattoo color over time by analyzing images of women who underwent NAC tattooing (*N* = 71) in a USA medical facility: the length of time is inversely correlated with scores and quantitative parameters of color and shape. The authors suggest that the fading phenomenon is predictable and measurable.

**Table 2:** Main characteristics and results of full-texts included in the review

Author; year; Country	Study design	Population	Methods					Adverse events	Main results
			Professionals and training	Time	Materials	Data collection tools/analysis	Endpoints		
Sowa et al. (2021) Japan	retrospective study	3D-E technique tattoo (*N* = 61) VS conventional technique tattoo (*N* = 49) after flap-based nipple reconstruction	One plastic surgeon with little training	21 months	Tattoo machine	10-points scale	Four senior plastic surgeons evaluated from pictures (1) 3D appearance (2) symmetry of NAC	NR	Higher appearance and symmetry in 3D-E tattoos.
Goh et al. (2011) UK	retrospective study	NAC tattoo (N patients = 172)	specialist nurse tattooist	8 years	Tattoo machine	5-points scale postal questionnaire	Patient satisfaction with the NAC	local erythema (*n* = 8); bleeding (*n* = 8); local infection (*N* = 4)	110 respondents; 70% were satisfied.
Starnoni et al. (2020) ITALY	restrospective study	NAC tattoo after nipple reconstruction (N patients = 48)	surgical trainees with 20 h- training with a professional tattoo artist	2 years	NR	Questionnaire	Patients’ satisfaction	scar nipple dehiscence (*N* = 3)	92% were satisfied or very satisfied.
DiCenso and Ficher-Cartlidge (2015) USA	cross-sectional study	BC RNs (*n* = 43) VS BC non-RNs (*n* = 25)	NA	5 weeks	NA	Online survey	likelihood of discussing options for NAC tattooing and recommending tattoo artists	NA	RNs were significantly less likely to recommend a professional tattoo artist than non-RNs
Cha et al. (2019) KOREA	retrospective study	Tattoo-only NAC reconstruction (*n* = 20) (subgroup of 95 patients that reconstructed the NAC with other techniques)	One surgeon Training: NR	9 months	Tattoo machine	10-points questionnaire	overall satisfaction	NR	Average overall satisfaction score: 8.1/10. significantly the highest compared with that of other techniques.
Aslam et al. (2015) UK	descriptive study	NAC tattoo after flap reconstruction (N patients = 292)	BC clinical nurse specialist trained in tattooing	9 years	NR	Questionnaire	(1) Patient satisfaction (2) Relationship between RT and tattoo fading	NA	Response rate: 60% 165/173 were happy with overall appearance 70% of RT patients reported fading.
Gava et al. (2020) ITALY	Pilot experimental study	BR (N patients = 169)	Advanced practice professionals	6 years	Dermograph	Phone interview	(1) Patient satisfaction (2) Service usefulness	minor complications (*N* = 3) (topical allergic reaction, abrasion, and soreness)	high satisfaction of the aesthetic results (90%); the service was useful/very useful (97%).
Smallman et al. (2018) AUSTRALIA	cohort study	Nurse-performed NAC tattoo (N patients = 169) VS plastic surgeon-performed tattoo (N patients = 111) after BR	Nurse/plastic surgeon Training: NR	6 years	Tattoo machine	BREAST-Q questionnaire	Before/after patient satisfaction	NR	Response rate: 48% Higher satisfaction with NAC after tattoo. No significant difference between clinicians.
Uhlmann, Martins and Piato (2019) BRAZIL	pilot experimental study	BR (N patients = 30)	Tattoo artist	22 months	Tattoo machine	5-point-scale	Patient and professionals’ satisfaction	No	Professionals: good and excellent overall esthetics (76%) and color (72%); tattooed patients (*N* = 20): overall satisfaction (95%) and color (100%).
Murphy et al. (2010) IRELAND	descriptive study	NAC tattooing after BR (N patients = 26)	clinical nurse specialist	2 years	Tattoo machine	(1) Phone survey (2) computer program	(1) Patient satisfaction (2) Color matching	No	Median satisfaction score: 4.6/5; mean color match of 91%.
Weissler et al. (2021) USA	retrospective study	NAC tattooing after BR (N patients = 539)	physician assistant or plastic surgery trained by a nurse	11 years	Tattoo machine	univariate analysis and multivariable regression model	identify risk factors for tattoo-related breast infections	tattoo-related infections ‘rate: 2.2%	85.7% of infections occurred in IBR patients RT and prepectoral IBR are independent predictors of tattoo-related breast infection.

*NAC* Nipplle Areola Complex; *BMI* Body Mass Index; *IBR* Implant Based Reconstruction; *IV* Intra-Venous; *RT* RadioTherapy; *NR* Not Reported; *NA* Not Applicable; *BR* Breast Reconstruction; *BC* Breast Cancer; *RN* Registered Nurse

**Table 3:** Main characteristics and results of only abstracts available included in the review

Author; year; Country	Study design	Population	Methods					Adverse events	Main results
			Professionals and training	Time	Materials	Data collection tools/analysis	Endpoints		
Rider et al. (2014) UK	Retrospective study	NAC tattoo after TRAM flap BR (*n* = 31) vs LD flap BR (n patients = 93)	Nurse practitioner Training: NR	8 years	NA	Fisher exact test	Any effects of reconstruction’s type and RT on N° of tattoo session	NA	No significant difference
Levites et al. (2014) USA	Retrospective study	NAC tattoo pictures (N patients = 71)	5 medical students Training: NR	6 years	NA	Customized scoring system software	Quantify color fading over time	NA	The fading phenomenon is predictable and measurable.

*TRAM* Transeverse Rectus Abdominus Myocutaneous; *LD* Latissimus Dorsi

### Synthesis of the results

This scoping review provides immediate implications for practice, adding knowledge on the impact on patient outcomes, professionals, and organizations, as well as on the type and quality of evidence available on the topic.

The NAC tattoo completes the reconstruction process after cancer treatment. Also, it represents a stand-alone option when surgical ones are not possible. These are the cases where a risk of poor outcome is consistent, like patients with fragile and/or tight breast skin or unsuitable for local skin flap elevation. Moreover, NAC dermopigmentation can be considered by those patients who refused to undergo another surgery for various reasons (exhaustion, fear, panic) [2]. The result is evaluated as satisfying and realistic [10], even if the projection is only apparent and not tridimensional.

Nevertheless, nipples' symmetry and projection are naturally challenged by the contralateral breast changes over time and by the skin flaps or RT consequences on the treated breast [6, 10]. Another disadvantage of the technique is the color's natural fading process, which requires periodical tattoo sessions to maintain the aesthetic result. The role of RT in this phenomenon is currently not completely clear: according to Aslam et al., tattoo fading is a significant problem in RT patients [25], but Rider et al. [24] suggest that the post-reconstruction RT exposition did not influence the aesthetic result. The fading phenomenon is often reported subjectively, although it may be quantifiable [29].

### The impact on professional competence

The professional training on the tattoo technique is specified in 6 papers. The issue remains variable and poorly described: in one study, a professional tattoo artist trained medical residents [27], while in another, a plastic surgeon trained a nurse [28]. Sowa et al. refined the tattoo technique, as it can be easily applied by medical staff instead of tattoo artists [10]. Nurses were the professionals primarily involved (N papers = 6), then medical staff (N papers = 4) and tattoo artists (N papers = 2). The figure of a clinical nurse specialist performing NAC tattooing was involved in 5 studies and worked in day-case or outpatient settings [17, 23–25].

### The impact on patient's health

The endpoints were mainly focused on the aesthetic result's satisfaction rate, which was favorable. Other Patient Reported Outcomes (PROs), like quality of life, sexual well-being, and other psychosocial issues, were evaluated by Smallman et al. with the BREAST-Q-specific module for breast reconstruction [28]; this study was the only one that used a validated questionnaire, and the only one that compared the results before and after the tattoo. The most frequent limitations declared in the studies reviewed were the poor evaluation criteria [2] and the use of study-specific questionnaires based on previous works [10, 11, 17].

### The impact on healthcare organizations

Studies described some experiences of nurse-led services [17, 23]. Several studies underline the necessity of these in-clinic services [2, 13], as NAC reconstruction represents a restoring and essential final step in the oncological care pathway [10, 17, 26]. Di Censo et al. describe the lack of standardization of tattoo services in the USA, suggesting that integrating a tattoo specialist into the healthcare team can ensure equal access to this care [26]. Even if a structured cost analysis did not emerge in the literature, some elements suggest significant savings: the Operating Room (OR) and general anesthesia are not required for the intervention, avoiding long-time healing and control visits for dressings. Gava et al. estimate about 1,600 euros of savings, considering costs for one patient NAC reconstruction (approximately 1 h of OR utilization, medical and nursing staff, recovery room, medical supplies, anesthesia, and pharmacy) [2].

## Discussion

According to the small body of literature, NAC tattooing represents a safe and well-tolerated technique that provides satisfactory aesthetic results. Additional research is needed to fill the knowledge gap on how this service may be provided and which outcomes it addresses. Despite the high risk of bias, some significant issues were extracted and analyzed. Further and larger studies are necessary to assess which is the best technique, the specific criteria according to which the tattoo-only approach is recommended, and eventual differences when compared with other procedures. Also, the relationship between fading and radiotherapy should be deeply understood with a more robust and broader design study. Specialist training pathways are necessary to define competencies and quality levels better. The NAC dermopigmentation needs to be provided by trained clinical specialists, aware of fragilities that can occur in women involved: low immune defenses, skin alterations, chemotherapy in progress, previous radiotherapy. Evaluating costs, some considerations about professional competence may deserve more attention: involving specialized nurses instead of medical staff may produce savings and reduce waiting lists for surgeries and medical check-ups. This ultimately translates into increased productivity and workers' motivation.

Furthermore, intercepting PROs with specific and validated instruments can provide reliable evidence about a large spectrum of effects on women's lives. Body image represents a multifaceted psychological experience comprising thoughts, beliefs, feelings, and attitudes. The related sufferings are perceived as global, and they should be identified from both a quantitative and qualitative point of view: the "Appearance-Pain" consists of the recomposed systematic view of the experimental indicators of suffering, linked to one of the dimensions of appearance [30]: this can represent a new complex outcome, that should be assessed with appropriate questionnaires, like the DAS59, also validated for breast cancer patients. Measuring the role of appearance may add more precision and personalization to cosmetic medicine, combining psychological and relational dimensions of the perceived body image. As the NAC dermopigmentation potentially implies all these aspects, it should not be considered an "extra" but part of the same restoring process involving mental and physical rehabilitation. Healthcare management is crucial in exploring the most feasible way to provide this service. Further research may explore the relationship between competence and patient outcomes: technical and artistic expertise should be combined with advanced competence in oncological care. Encouraging guides on the training curricula in medical tattooing are developing [31]: assessing the effective integration of all the competence elements ensures the highest quality service and patient safety. Finally, future studies might focus on a specific instrument development: validated measures assessing appearance concerns of the NAC among perioperative care will quantify the impact of NAC tattooing, more systematical and objective, filling a current knowledge gap.

## Limitations

The present review has some limitations: first, only English papers were included, which may have partialized the results. Secondly, the poorness of the literature available, both in the number of studies available and in the methodological quality, cannot permit the generalization of results.

## Conclusions

The tattooing of the Nipple-Areola Complex is a safe and satisfactory intervention that can finally restore the integrity of appearance for women who have faced demolitive cancer treatments, with an auspicable improvement of self-perception, anxiety, depression, sexual function, and identity role. Future studies are urgently needed to deeply understand the relationship with quality of life outcomes and assess the processes involved in this service, particularly the feasibility of nurse-led services. It appears worth exploring the appropriate measurements and research methodology for this intervention for women’s health.
